# Comparative Outcomes of Percutaneous and Conventional Open Pedicle Screw Fixation for Single-level Thoracolumbar Spine Injury: Randomised Controlled Trial

**DOI:** 10.5704/MOJ.2403.014

**Published:** 2024-03

**Authors:** K Choovongkomol, U Piyapromdee, S Thepjung, T Tanaviriyachai, S Jongkittanakul, W Sudprasert

**Affiliations:** Department of Orthopedic Surgery, Maharat Nakhon Ratchasima Hospital, Nakhon Ratchasima, Thailand

**Keywords:** spinal fracture, thoracolumbar spine, pedicle screws, minimal invasive surgery

## Abstract

**Introduction:**

To compare post-operative outcomes of percutaneous pedicle screw fixation (PPSF) vs open pedicle screw fixation (OPSF) in patients with thoracolumbar spine fractures with no neurological deficits.

**Materials and methods:**

In a randomised controlled trial, patients received short-segment fixation with intermediate screws. We assessed post-operative back pain (Visual Analog Scale or VAS), blood loss, operative/fluoroscopy times, radiographic parameters, and oswestry disability index (ODI) scores at 1, 2, 3, 6, 9, and 12 months.

**Results:**

Between January 2018 and October 2019, 31 patients received PPSF and 30 OPSF. Mean intra-operative blood loss was 66.45 (±44.29) ml for PPSF vs 184.83 (±128.36) ml for OPSF (p<0.001). Fluoroscopy time averaged 2.36 (±0.76) minutes for PPSF vs 0.58 (±0.51) minutes for OPSF (p<0.001). No significant differences existed in operative time or post-operative VAS scores. Radiographic parameters (kyphosis angle and vertebral height ratios) didn't significantly differ post-operatively or at 12 months. However, ODI scores differed significantly at 6 months (p=0.025), with no difference at 12 months.

**Conclusion:**

In this trial, PPSF was comparable to OPSF in improving ODI scores at 12 months but showed earlier improvement at 6 months and reduced blood loss. Radiographic outcomes remained similar between groups over 12 months.

## Introduction

Thoracolumbar spine fractures are a common issue in the field of spinal trauma, especially in the biomechanically transitional zone known as the thoracolumbar junction (T10-L2). These injuries typically result from high-energy traumas like traffic accidents or falls from significant heights. To classify these fractures and determine spinal stability, the three-column concept introduced by Denis has been widely employed^1,2^. At present, the choice of treatment for these fractures’ hinges on several factors, including the fracture type, mechanism of injury, neurological status, and the presence of posterior ligamentous complex injuries that may necessitate surgical intervention. Additionally, classification systems like the Thoracolumbar Injury Classification and Severity Score (TLICs) and the AO Spine thoracolumbar spine injury classification system (TL-AOSIS) are available to aid surgeons in making informed decisions^3-5^.

Surgical treatment is often the preferred choice for patients with thoracolumbar fractures due to its superior therapeutic outcomes compared to conservative approaches like bed rest and immobilisation^6^. Traditionally, open posterior pedicle screw fixation (OPSF) has been the standard method for addressing thoracolumbar spine fractures. However, conventional open procedures come with significant drawbacks, including substantial blood loss, a high risk of infection, prolonged post-operative pain, and disability^7^. In an effort to mitigate perioperative complications, Magerl introduced a pioneering minimally invasive treatment approach for thoracolumbar fractures. This innovative technique involves the use of external fixators and percutaneous pedicle screws^8^. Its primary goal is to preserve the paravertebral musculature and minimise damage to the zygapophysial joint^9^. Percutaneous pedicle screw fixation (PPSF) offers several advantages, such as reduced intra-operative blood loss and shorter operative duration. Studies have also demonstrated that patients who undergo PPSF experience superior post-operative pain relief, as indicated by the visual analogue pain scale^10-14^. Due to these promising outcomes, PPSF has gained increasing popularity as a preferred method for the fixation of thoracolumbar spine fractures.

Regarding fixation constructs, employing short segment posterior pedicle screw fixation (involving one level above and below the fractured vertebra) holds distinct advantages as it preserves spinal motion and reduces intra-operative morbidity. It's worth noting that while long segment posterior fixation may offer improved radiographic outcomes compared to short segment fixation, it's essential to recognise that short segment fixation alone can still lead to favourable clinical results^15,16^. Notably, when short segment fixation includes the fractured vertebra, the radiographic outcomes are comparable to those achieved with long segment fixation^17^.

While numerous meta-analyses have explored percutaneous pedicle screw fixation (PPSF) and yielded promising results, there remains a shortage of randomised controlled trials and limited reports that specifically delve into functional outcomes. Consequently, the primary aim of this randomised controlled trial is to evaluate the post-operative radiographic and functional outcomes of PPSF when compared to open posterior pedicle screw fixation (OPSF) in patients with single-level thoracolumbar spine fractures and no neurological injury.

This study is designed to offer valuable insights into the effectiveness of PPSF in enhancing both radiographic and functional outcomes within this particular patient group.

## Materials and Methods

This study was a prospective randomised controlled trial designed to compare the outcomes of percutaneous pedicle screw fixation (PPSF) and open pedicle screw fixation (OPSF) for single-level thoracolumbar spine injuries, following the CONSORT 2010 guidelines^18^. The study had received ethics approval from the Institutional Review Board of the author's affiliated institution.

In this study, our inclusion criteria comprised patients aged 18-60 with single-level thoracolumbar spinal injuries who did not exhibit neurological deficits and had a Thoracolumbar Injury Classification and Severity Score (TLICs) of ≥4. We excluded patients with spinal anatomical variations or deformities, a history of neurological dysfunction or mental illness, those who declined to provide informed consent, individuals with coagulation disorders, those who had used aspirin or NSAIDs within the last seven days, and patients with multiple traumas. Upon obtaining written informed consent from the patients, we enrolled them and then randomly divided them into two groups: percutaneous pedicle screw fixation (PPSF) and open pedicle screw fixation (OPSF). The randomisation process involved blocks of four, each containing a computer-generated random sequence of an equal number of the two treatments. In the operating room, before the surgery, the surgeon opened an opaque envelope to reveal the assigned treatment group for each patient. It's important to note that both the patients and the surgeons were aware of the assigned treatments, so blinding was not applied in this trial. The surgeries were performed by a single experienced spine surgeon from our institute.

All patients underwent short segment fixation utilising an intermediate screw configuration, which included screws placed cranially, caudally, and at the fractured vertebra. To achieve indirect reduction, patients were positioned in a hyperextended prone posture. In the case of conventional open pedicle screw fixation (OPSF), a posterior midline approach was employed. The global standard screw (GSS) monoaxial pedicle screw system [GS Medical Co., Ltd., Geumcheon-gu, Seoul, Korea] was utilised following conventional procedures. Pedicle screws were sequentially inserted using a freehand technique, with screw positions verified through both anteroposterior and lateral fluoroscopy views. Subsequently, two appropriately sized and curved rods were inserted.

**Fig 1:**
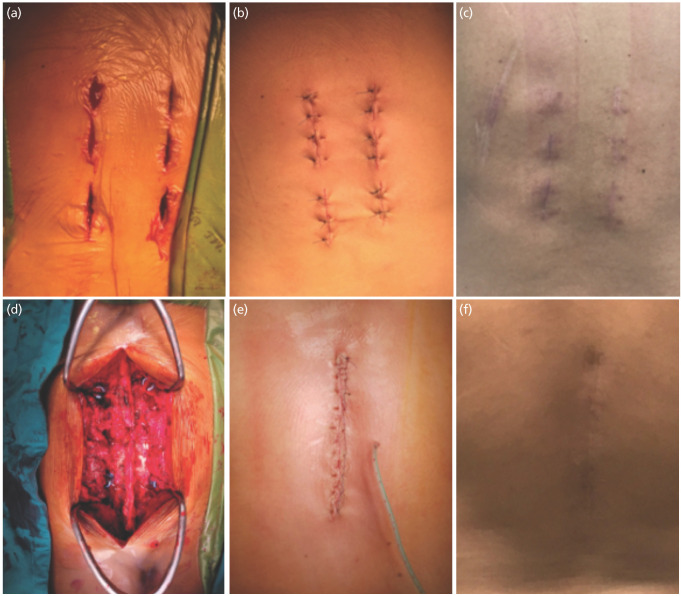
(a-c) The surgical incisions and wound of patients who underwent percutaneous pedicle screw fixation and (d-f) open pedicle screw fixation.

In percutaneous pedicle screw fixation (PPSF), the Aesculap S4 element MIS system [Aesculap, a B.Braun company, 78532 Tuttlingen, Germany] pedicle screws were inserted under continuous fluoroscopy guidance throughout the procedure. A Jamshidi trocar stylet was introduced through the skin and fascial incisions and positioned at the surface corresponding to each pedicle's projection area. When the trocar reached the bony surface of the pedicle on the lateral view, its tip was situated at the lateral margin of the pedicle on the anteroposterior view. With continuous fluoroscopic monitoring, the trocar was advanced through the pedicle and into the vertebral body. Following stylet removal, a guidewire was threaded through the trocar and advanced into the vertebral body under lateral view. Dilators were employed to create space between the fascia and muscles. Cannulated pedicle screws were then inserted into the vertebral bodies through the guidewires. Two rods of suitable lengths and curvature were introduced through the lower incision and threaded sub-fascially into the remaining screw heads, subsequently secured using cranial bolt heads. It's noteworthy that posterior spinal fusion was not performed in either of the procedures conducted during this study (Fig. 1).

**Fig 2:**
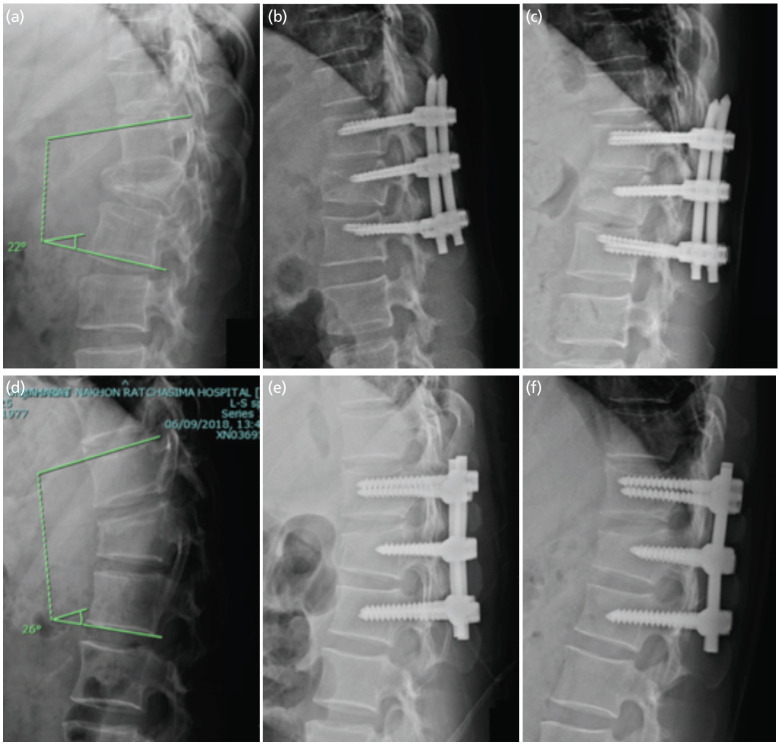
(a-c) Illustrates the initial, post-operative, and one-year follow-up radiographs of patients who underwent percutaneous pedicle screw fixation and (d-f) open pedicle screw fixation.

Patients in both study groups received routine prophylactic antibiotics and effective pain management. Furthermore, they were encouraged to commence early ambulation without the use of thoracolumbar orthosis. However, to ensure proper healing and recovery, all strenuous and heavy activities were strictly prohibited for a duration of three months. Upon discharge from the hospital, patients underwent regular clinical and radiological evaluations at the orthopaedic outpatient clinic at intervals of 1, 2, 3, 6, 9, and 12 months (Fig. 2). The collected data encompassed a range of factors, including pain assessed via the visual analogue scale (VAS), patient age and gender, the cause of injury, fracture type classified under the AO-OTA system, the level of injury, intra-operative blood loss, operative duration, time to surgery, and length of hospital stay. Additionally, we gathered information on the thoracolumbar injury classification and severity scores (TLICs) and McCormack's scores from radiographs and CT scans.

For the purpose of our analysis, the reference anterior and posterior vertebral heights were defined as half of the sum of the heights of the vertebra immediately above and below the fractured vertebra. We calculated the anterior and posterior fractured vertebral height percentages as the ratio of the fractured vertebra's height to the reference vertebral height. To determine the Cobb angle, we measured the angle between the superior endplate of the upper vertebra and the inferior endplate of the lower vertebra of the fractured segment. The correction value was derived by subtracting the immediate post-operative measurement from the pre-operative measurement, while the correction loss value was calculated by subtracting the immediate post-operative measurement from the final follow-up measurement for all parameters. Functional outcomes were assessed using the Oswestry Disability Index (ODI) functional score.

The determination of our sample size was based on the ODI scores obtained at the final follow-up in a study conducted by Wang *et al*^12^. In that study, they reported ODI scores of 6.1±3.5 for patients who underwent OPSF and 3.7±2.1 for those who received PPSF. Through our calculations, we established that a sample consisting of 28 patients in each treatment group would provide a statistical power of 0.85 with a two-tailed significance level of 0.05, enabling us to detect any significant differences between the groups. To ensure robustness and prevent potential data loss due to patient follow-up discontinuation, we made the decision to increase the sample size to 32 patients per treatment group.

All data analyses were performed on an intention-to-treat basis, which means that all patients were included in the analysis according to the groups to which they were initially randomised. For continuous outcomes, we presented the results by describing the means and standard deviations for each group. Categorical outcomes were presented by indicating the frequencies and percentages for each group. Statistical analyses were carried out using STATA version 16.0 [StataCorp, College Station, TX, USA]. Two-sided significance tests were used, with a significance level set at p-value > 0.05. Fisher's exact test was employed for the analysis of categorical data, while the student's t-test was used for continuous data.

## Results

**Fig 3:**
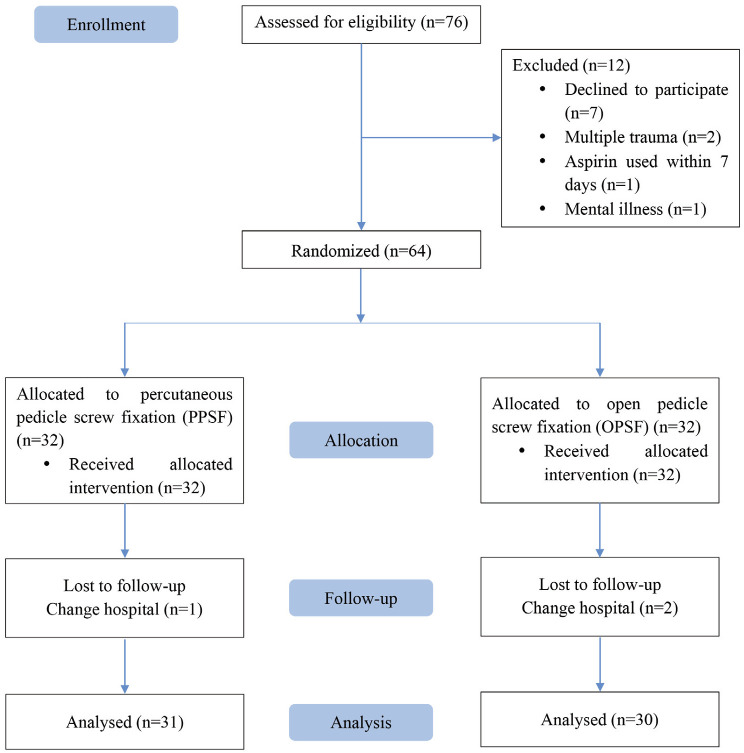
CONSORT study flow diagram.

Between January 2018 and October 2019, a total of 76 patients were initially considered eligible for inclusion in the study. However, we excluded 12 patients for various reasons. Seven patients chose not to participate, two had multiple injuries, one had used aspirin within seven days prior to the procedure, and one had a history of mental illness. We had 32 patients in the PPSF group and 32 in the OPSF group. Following discharge from the hospital, we unfortunately lost track of one patient from the PPSF group and two from the OPSF group (Fig. 3). Our analysis revealed no significant differences between the two groups in terms of demographic characteristics. This included factors such as fracture type, the level of injury, time to operation, TLICs, and McCormack scores (Table I).

**Table I:** Patients characteristics and demographic data.

Demographic data	PPSF	OPSF	p-value
	(N = 31)	(N = 30)	
Age	44.48 (±12.73)	39.3 (±12.50)	0.114
Gender			
Male	16 (52%)	20 (67%)	0.300
Female	15 (48%)	10 (33%)	
Cause of injury			
Fall from height	17 (55%)	16 (54%)	1.000
Traffic accident	13 (42%)	13 (43%)	
Body assault	1 (3%)	1 (3%)	
Fracture type (AO-OTA)			
A3	9 (29%)	15 (52%)	0.176
A4	19 (61%)	10 (35%)	
B1	2 (6%)	3 (10%)	
B2	1 (3%)	1 (3%)	
Level of injury			
T11,12	3 (10%)	7 (23%)	0.149
L1	23 (74%)	14 (47%)	
L2	5 (16%)	7 (23%)	
L3	0 (0%)	2 (7%)	
Time to operation (day)	6.23 (±4.11)	7.1 (±3.64)	0.384
TLICs	4.61 (±0.72)	4.59 (±0.95)	0.902
McCormack’s score	5.74 (±1.24)	5.52 (±1.06)	0.454

**Fig 4:**
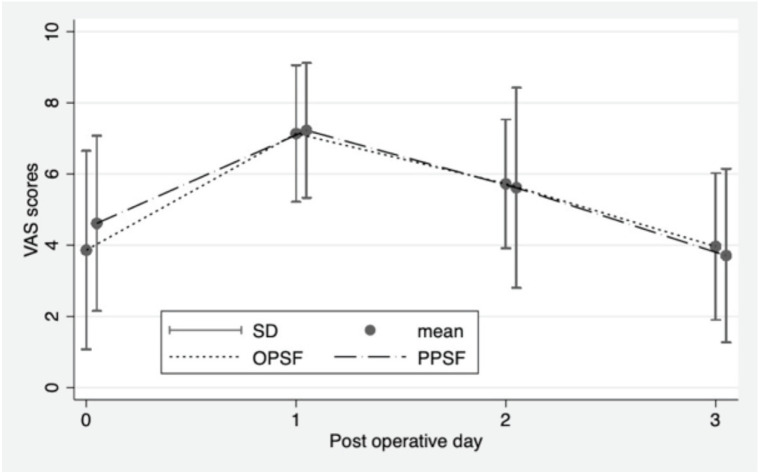
Comparative post-operative visual analogue pain score of percutaneous pedicle screw fixation (PPSF) and open pedicle screw fixation (OPSF).

There were no significant differences observed between the two groups in terms of operative time and length of hospital stay. However, a notable discrepancy was found in intra-operative blood loss, with the PPSF group experiencing a significant reduction when compared to the OPSF group (66.45±44.29ml versus 184.83±128.3ml p<0.001). Similarly, the mean haematocrit decrease was significantly lower in the PPSF group (2.35±1.47%) compared to the OPSF group (4.85±2.49%) (p=0.001). In terms of fluoroscopic time, the PPSF group had significantly longer durations (p<0.001). When it comes to post-operative VAS scores, there were no significant differences between the two groups on the first, second-, and third days following surgery (Table II, Fig. 4). Importantly, we did not observe any wound complications, infections, implant failures, or neurological injuries resulting from the procedures conducted during this study.

**Table II:** Perioperative outcome and visual analogue pain score of percutaneous pedicle screw fixation (PPSF) and open pedicle screw fixation (OPSF).

Outcomes	PPSF (N=31)	OPSF (N=30)	p-value
Operative time (mins)	62.00 (±14.20)	63.45 (±16.81)	0.719
Intra-opertive blood loss (ml)	66.45 (±44.29)	184.83 (±128.36)	0.000
HCT decrease (mg%)	2.35 (±1.47)	4.85 (±2.49)	0.001
Fluoroscopic time (mins)	2.36 (±0.76)	0.58 (±0.51)	0.000
Length of stay (day)	11.71 (±4.68)	13.57 (±4.26)	0.111
VAS scores			
Pre-operative	4.61(±2.46)	3.86(±2.79)	0.272
Post-operative day 1	7.23(±1.89)	7.14(±1.92)	0.859
Post-operative day 2	5.61(±2.81)	5.72(±1.81)	0.857
Post-operative day 3	3.71(±2.44)	3.97(±3.18)	0.664

There were no significant differences observed in the correction of kyphosis angle or the reduction of anterior and posterior vertebral height between the two groups (p>0.05). In terms of screw malposition, it was found in 7 out of 186 cases (3.76%) in the PPSF group, compared to 4 out of 180 cases (2.22%) in the OPSF group (p=0.509). Importantly, none of the patients required revision surgery due to screw malposition. At the one-year follow-up, there were no significant differences in terms of kyphosis progression or anterior vertebral height (AVH) ratio loss correction (p>0.05). However, it's noteworthy that the loss correction in posterior vertebral height (PVH) was significantly higher in the OPSF group (5.87±6.13) than in the PPSF group (2.67±6.05) (Table III).

**Table III:** Radiographic outcomes of percutaneous pedicle screw fixation (PPSF) and open pedicle screw fixation (OPSF).

Radiographic parameters	PPSF (N = 31)	OPSF (N= 30)	p-value
Screw malposition (screws)	7/186 (3.76%)	4/180 (2.22%)	0.509
Kyphosis angle			
Pre-operative kyphosis	18.03 (±7.64)	16.07 (±9.57)	0.382
Kyphosis correction	11.87 (±6.31)	12.10 (±5.54)	0.880
Post-operative kyphosis	6.16 (±6.06)	3.97 (±8.87)	0.265
One-year follow-up	10.94 (±6.78)	8.93 (±9.88)	0.358
Kyphosis progression	4.74 (±4.88)	5.20 (±4.33)	0.700
Anterior Vertebral Height Ratio (%)			
Pre-operative	55.39 (±14.42)	56.74 (±16.53)	0.747
AVH Ratio reduction	20.79 (±10.69)	21.64 (±17.63)	0.821
Post-operative	76.18 (±13.16)	78.31 (±13.13)	0.530
One-year follow-up	71.92 (±13.19)	72.80 (±14.83)	0.806
AVH Ratio loss correction	4.26 (±7.95)	5.51 (±7.06)	0.520
Posterior Vertebral Height Ratio (%)			
Pre-operative	87.32 (±10.59)	86.88 (±18.66)	0.910
PVH Ratio reduction	1.38 (±6.01)	4.75 (±15.64)	0.268
Post-operative	88.69 (±8.18)	91.62 (±8.38)	0.172
One-year follow-up	86.02 (±6.78)	85.75 (±8.57)	0.892
PVH ratio lost correction	2.67 (±6.05)	5.87 (±6.13)	0.045

**Fig 5:**
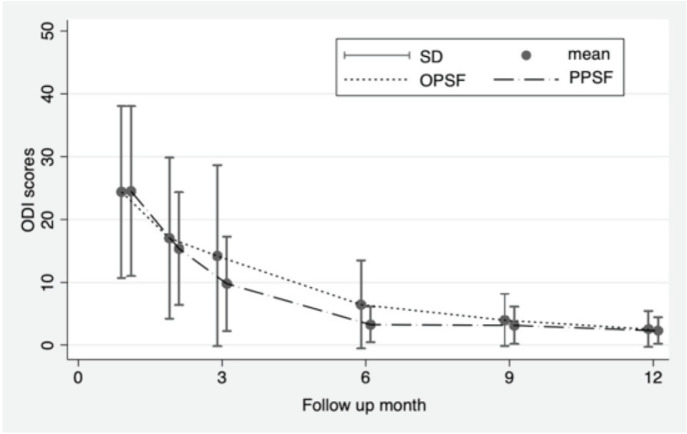
Comparative oswestry disability index (ODI) functional score between percutaneous pedicle screw fixation (PPSF) and open pedicle screw fixation (OPSF).

Both surgical techniques led to a significant improvement in the Oswestry Disability Index (ODI) functional score over the follow-up period. Notably, there were no significant differences between the two groups at 1, 2, 3, 9, and 12 months after the operation. However, it's worth highlighting that at the six-month mark post-operation, there was a significant difference in the ODI score. Specifically, the ODI score was 6.48±7.05 in the OPSF group and 3.29±2.85 in the PPSF group (p=0.025) (Table IV, Fig. 5).

**Table IV:** Oswestry disability index (ODI) functional score of PPSF and OPSF.

ODI functional score	PPSF (N=31)	OPSF (N=30)	p-value
1 month	24.51±13.50	24.41±13.66	0.977
2 months	15.38±8.96	17.05±12.78	0.609
3 months	9.81 ±7.51	14.07±14.53	0.142
6 months	3.29±2.85	6.48±7.05	0.025
9 months	3.17±3.00	4.00±4.20	0.491
12 months	2.32±2.14	2.57±2.91	0.723

## Discussion

The conventional open procedure has long been the standard for posterior spinal fusion or fixation. However, the field of minimally invasive spinal surgery has emerged as a response to mitigate the morbidity associated with traditional approaches. Conventional methods often involve detaching paraspinal muscle insertions and subjecting muscles and ligaments to prolonged traction, which can potentially result in muscle denervation, scarring, and adverse effects on post-operative trunk muscle performance^9,19^.

PPSF has demonstrated several advantages over conventional open surgery, which include reduced bleeding, decreased post-operative pain, and shorter operative and hospitalisation times^10,14,20,21^. In this study, PPSF notably reduced intra-operative blood loss and haematocrit decrease. However, there were no significant differences observed in hospital stays and operative time, possibly because the surgical team was more experienced with OPSF. Consistent with recent reports, we found no significant disparity between the two groups in terms of post-operative pain, as measured by the visual analogue scale (VAS)^12^. It's important to recognise that thoracolumbar spine injuries typically involve high-energy mechanisms, resulting in extensive soft tissue damage prior to surgery. Consequently, patients in both groups commonly experience significant levels of pain. While Phan *et al* reported a reduced risk of wound infection with PPSF, our study did not find a significant difference in this regard^10^. It should be noted that PPSF did require a significantly longer fluoroscopic time, with an average of 2.36 minutes per case or 0.39 minutes per screw, which is consistent with other reports. It's important to be aware that radiation exposure is a notable drawback of the percutaneous technique^22-24^.

Regarding radiographic outcomes, both groups exhibited significant improvements in kyphosis angle and anterior and posterior vertebral body ratios compared to their pre-operative parameters. These findings align with previous studies, which have reported that percutaneous techniques are as effective as conventional methods in restoring vertebral body height and improving kyphosis angle^10,20,21,25-27^. We did observe screw malposition in both groups, with rates of 3.76% in the PPDS group and 2.22% in the OPDS group, although this difference was not statistically significant. Over the course of a one-year follow-up, both groups experienced a minor loss of correction, which was consistent with previous reports^12,24,28^, and there was no statistically significant difference between them. It's worth noting that although OPSF did show a statistically significant loss of correction in posterior vertebral body height ratio compared to PPSF, this difference did not have a clinical impact. Despite neither group undergoing posterior fusion, the radiographic outcomes were deemed acceptable.

We observed similar improvements in functional scores at various time points (1, 2, 3, 9, and 12 months) after surgery in both groups. However, at the six-month mark, posterior percutaneous screw fixation (PPSF) demonstrated superior results. This finding is consistent with a study by Wang *et al*, which also noted a similar pattern of functional improvement^29^. Additionally, Tu *et al* reported that functional outcomes were better in the PPSF group at three months but comparable to the OPSF group at the six-month follow-up^30^. The minimally invasive approach, which results in less soft tissue damage, likely contributes to a faster recovery compared to the conventional open method. Ultimately, both techniques were effective in achieving favourable functional outcomes at the final follow-up.

This study had several strengths. It focused on neurologically functional patients, and a single surgeon conducted a randomised controlled trial, ensuring consistency in surgical technique. We collected both radiographic and functional outcome data, providing a comprehensive assessment. Moreover, all patients received the same six-screw construct, including an intermediate screw, which aimed to preserve spine motion and offer stability. However, the study had limitations. The sample size was relatively small, which might reduce the reliability of the findings. Additionally, the relatively short follow-up period was influenced by some patients opting to remove their implants after 12 months, potentially impacting the outcomes. Consequently, we chose to analyse our data specifically at the 12-month mark. To generalise our findings, conducting further multicentre studies is imperative.

## Conclusion

In this randomised controlled trial, we didn't observe clear superiority of PPSF over OPSF in terms of improving function, as measured by the ODI, at the 12-month follow-up. However, PPSF did show earlier improvement in function at the six-month mark, along with a notable reduction in intra-operative blood loss. Importantly, radiographic outcomes were similar in both treatment groups throughout the 12-month follow-up period. As a result, PPSF can be considered an excellent option for treating single-level thoracolumbar spine injuries in patients with intact neurological function.
